# Age dependent associations of risk factors with heart failure: pooled population based cohort study

**DOI:** 10.1136/bmj.n880

**Published:** 2021-04-01

In this paper by Jasper Tromp and colleagues (BMJ 2021;372:n461, doi:10.1136/bmj.n461), the first and last names of author Ramachandran S Vasan were the wrong way round. The online version has been amended.

